# Reversible hypothyroidism and Whipple's disease

**DOI:** 10.1186/1472-6823-6-3

**Published:** 2006-05-10

**Authors:** Huy A Tran

**Affiliations:** 1 Hunter Area Pathology Service, John Hunter Hospital, Locked Bag Number 1, Hunter Mail Region Centre, New South Wales, 2310, Australia

## Abstract

**Background:**

The major cause of primary hypothyroidism is autoimmune mediated with progressive and permanent destruction of the thyroid gland resulting in life-long replacement therapy. Treatable and reversible hypothyroidism is unusual and here forth is such a case due to infection of the thyroid gland with Tropheryma whippleii, Whipple disease.

**Case presentation:**

A 45 year-old female presented with symptoms and signs consistent with primary hypothyroidism, which was also confirmed biochemically. Her response to thyroxine replacement therapy was poor however, requiring a significantly elevated amount. Further investigation revealed the presence of Whipple's disease involving the gastrointestinal trace and possibly the thyroid gland. Her thyroxine requirement decreased drastically following appropriate antimicrobial therapy for Whipple's disease to the extent that it was ceased. Thyrotropin releasing hormone testing in the steady state suggested there was diminished thyroid reserve due to Whipple's disease.

**Conclusion:**

This is the first ante-mortem case report studying the possible involvement of the thyroid gland by Whipple's disease. Despite the normalization of her thyroid function test biochemically after antibiotic therapy, there is diminished thyroid reserve thus requiring close and regular monitoring.

## Background

A 45-year-old female presents with a 6-month history of progressive tiredness, lethargy and mild, intermittent alopecia. She also reported mild shortness of breath and ankle swelling. Her family has noted a slowed mentation with approximately 8 kg weight loss (~10% of body weight) during this time. There was no fever, diarrhea, abdominal pain, malaise or arthralgia on systemic review. She has never smoked and there was no significant past history. No first-degree relative had any history of thyroid disease.

## Case presentation

Clinically she appeared tired and lethargic and myxedematous looking with a body mass index of 28 kg/m^2 ^(height of 1.58 m and weight 71 kg). She was oriented, coherent and afebrile. Her vital signs were stable. There was small and non-tender goiter and her general examination was normal. There was no generalized lymphadenopathy. Thyroid ultrasound showed a mildly enlarged gland with volume of 12 mls (reference range (RR), 6–10)[1], without any nodule. A 24-hour radioactive iodine showed heterogenous patchy uptake and distribution at 15% (RR: 10–36). Her laboratory investigations are listed in Table 1.

**Table 1:** Laboratory Parameters. (Numbers in parentheses represent the reference ranges.)

Haemoglobin (115–165 g/L)	94
White cell count (4.0–11.0 × 10^9 ^per L)	12.3
Platelet count (150–400 × 10^9 ^per L)	578
Erythrocyte Sedimentation Rate (<10 mm/hr)	38
Sodium(137–143 mmol/L)	138
Potassium (3.5–5.5 mmol/L)	3.4
Urea (3.6–6.8 mmol/L)	3.1
Creatinine (0.06–0.10 mmol/L)	0.05
Glucose (random) (3.0–7.7 mmol/L)	3.2
Calcium (2.18–2.50 mmol/L)	2.11
Ionised Calcium (1.04–1.24 mmol/L)	0.95
Phosphate (0.86–1.36 mmol/L)	0.88
CK (<120 U/L)	110
Total Cholesterol (<4.0 mmol/L)	3.9
Triglyceride (<1.8 mmol/L)	1.5
Iron (10–27 umol/L)	5
Ferritin (30–260 ug/L)	19
Transferrin (2.0–3.8 g/L)	8.4
Total iron binding capacity (48–68 umol/L)	77
Albumin (35–42 g/L)	28
Total protein (67–79 g/L)	55
TSH (0.4–4.0 mU/L)	88.4
Free tetra-iodothyronine (10.5–26.5 pmol/L)	4.3
Anti-thyroglobulin antibody titre (<1:100)	<1:100
Anti-thyroperoxidase antibody titre (<1:100)	<1:100
TSH Stimulating Immunoglobulin (<10 U/mL)	< 10

Levo-thyroxine was initiated but with very little improvement in her symptoms despite assured compliance. At 6 weeks' review, her thyrotropin (TSH) remained at 75 mU/L on 100 μg of L-thyroxine daily. The dose was adjusted according to clinical symptoms and TSH values every 6–8 weeks. The latter was 66 mU/L on 200 μg of L-thyroxine, 52 mU/L on 300 μg and 58 mU/L on 400 μg daily. Finally, she was maintained on 500 μg daily with steady state TSH of 24.5 mU/L despite moderate improvement in symptoms only. Malabsorption then became evident given her biochemical parameters (Table 1) and the 1 mg-thyroxine absorption test confirmed such suspicion. Her fT4 concentrations rose modestly. Levels were 5.6, 7.8 and 8.1 pmol/L at 0, 2 and 4 hours respectively. Her anti-transglutaminase antibody was normal, ruling out Coeliac's disease. As her symptoms persisted, an upper gastrointestinal endoscopy was performed which revealed, surprisingly, a normal duodenal mucosal appearance. Unfortunately, enteroscopic photography was not available.

### Diagnostic procedures for Whipple's disease (WD)

**Figure 1:**
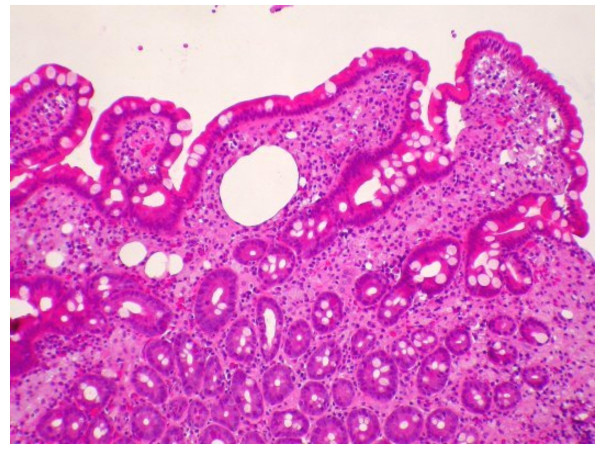
Histology of the duodenal biopsy showing clubbed epithelial villi with inflammatory infiltrate including numerous foamy macrophages (haemtoxylin-eosin stain, magnification × 250).

**Figure 2:**
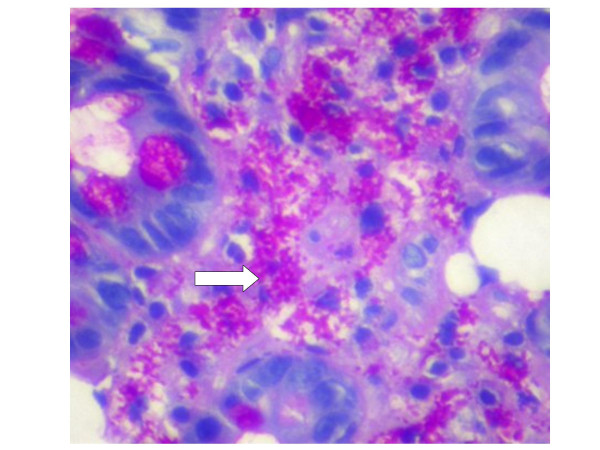
Histology of the same biopsy at high-power magnification using PAS stain, showing foamy macrophages containing the characteristic rod-shaped inclusion bodies (arrow).

**Figure 3:**
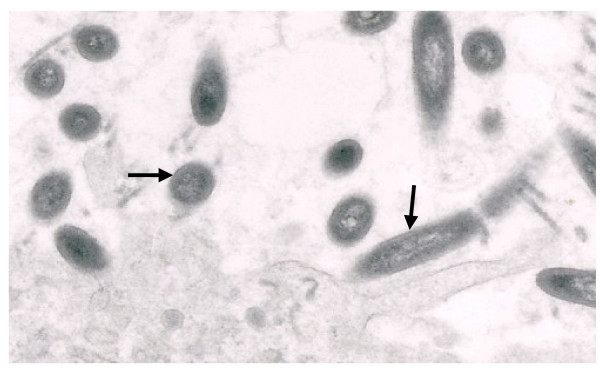
Electron microscope of the duodenum tissue, demonstrating the typical appearance of TW organism. The bacteria (arrows) appear rod-shaped and consisted of a thick cell wall and granular-fibrillar cytoplasm.

Histology of the biopsy the second part of the duodenum revealed the typical appearance of WD including clubbed epithelial villi and foamy macrophages, which were diastase Periodic acid-Schiff (PAS) positive, infiltrating the lamina propia (Figures 1 &2). Immunohistochemistry was not available. WD was further confirmed by electron microscopy (EM), (Figure 3), and positive polymerase chain reaction (PCR) for TW in both duodenal tissue and peripheral circulating leucocytes. Culture for atypical mycobacterium from the biopsied tissue, as part of a negative control, was negative. PCR was done using specific primers W3AF and W4AR established previously by Ramzan et al. [2]. Negative controls included normal duodenal tissue (PAS negative) from 2 patients and DNA from E. Coli. The result yielded a specific amplicon, 160 base pair in size, analysed by agarose gel electrophoresis, stained with ethidium bromide and visualized under UV light. Both strands of the PCR product demonstrated sequence homology with the TW 16S ribosomal RNA gene sequences deposited in GenBank and EMBL databases by means of the University of Wisconsin Genetic Computer Group software package (Madison, Wisconsin).

Two attempts at thyroid cytology aspirations were performed to identify the presence of TW. Unfortunately, both failed to extract adequate DNA to perform the identification of 16S rDNA by PCR amplification and sequencing. Cytological examination showed the presence of cellular inflammatory infiltrates including a small number of lymphocytes, which were PAS-negative.

### Progress

The patient was diagnosed with WD and treated with Trimethoprim-Sulphamethoxazole (Bactrim) during which she improved steadily and regained in reasonable health in the following 6 months. She was able to return to work as an assistant librarian, albeit part-time. A formal neurological assessment was not performed including cerebral magnetic resonance imaging and cerebral spinal fluid examination.

She then developed tremor and palpitation and her TSH was found to be suppressed mandating thyroxine cessation. The patient remained off thyroxine and follow-ups at 6 and 12 months showed a TSH of 2.6, 1.5 mU/L with fT4 of 15.8 and 20.5 pmol/L respectively.

**Figure 4:**
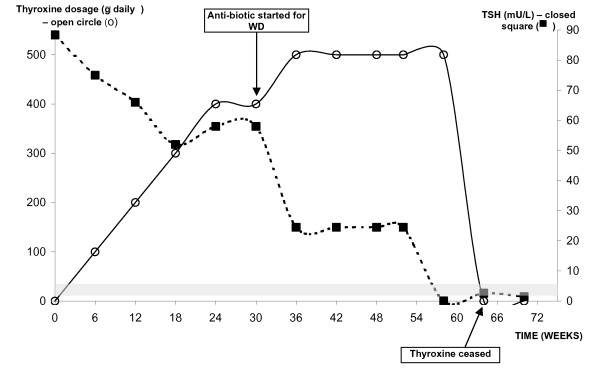
The relationship between TSH and thyroxine dosage during the course of treatment with oral antibiotic. The shaded area represents the TSH reference range.

At the end of 12 months of antibiotic therapy, all her laboratory parameters improved. She regained ~5 kg although the patient was conscious of not regaining too much weight. At that time, her TSH and fT4 levels were 3.2 mU/L and 19.5 pmol/L respectively. Her TSH levels in response to various thyroxine doses are illustrated in Figure 4. Her thyroid auto-antibodies status was unchanged. Her repeat PCR on peripheral blood was negative for TW. At 18 months, as there were concerns regarding thyroid reserve and despite the normalization of her thyroid parameters, an intravenous Thyrotropin Releasing Hormone (TRH) stimulation test was performed. TSH levels rose from 1.8 to 89.8 and 65.0 mU/L at 0, 30 and 60 minutes respectively.

Long term follow-up included close monitoring of her thyroid status. A repeat of her TRH stimulation test was performed at 36 months was near identical to previous. No repeat endoscopy and small bowel biopsy were performed as clinical progress was satisfactory.

WD was historically considered a primarily gastrointestinal disease with diarrhoea being the predominant symptom. However, it is now widely accepted as a generalized systemic disease involving many organs. The case report is atypical in that patient concerned is female with few gastrointestinal symptoms [3,4]. The diagnostic procedures available for the diagnosis of WD include small bowel biopsy (SBB), EM findings and PCR. The appearance of the SBB and relevant stainings are non-specific [5]. EM, which some considered diagnostic, is time consuming and is not routinely available. Similarly, PCR is useful but is technically challenging. To further confound the issue, TW detection using PCR has been found in salivary content of healthy subjects from England [6], sewage treatment plants [7] and gastric juice and duodenum of patients with no clinical signs or suspicion of WD [8]. Thus these three tools are non-specific on their own but a combination of any two positive results can contribute to the diagnosis [5]. Immunohistochemistry, staining for the PAS containing granules in the bacteria, is pivotal in the diagnosis. Cross-reaction is very low (with Shigella and Streptococcus agalactiae) and thus is very useful [9]. However, this test is highly specialized, not readily and routinely available due to the rarity of the disease and hence could not be performed. Recently, culture of the organism has become possible but with great difficulties due to the organism's slow growth with the calculated doubling time of 18 days. Therefore this great advance remains mostly in the research setting [5,10]. Where possible, culture for organisms that can mimic the histological findings of WD, such as Mycobacterium tuberculosis, should be carried out as the results will heavily influence the choice of antimicrobial therapy [4]. Thus, due to restricted availability of diagnostic tests, the diagnosis relies heavily on the combination of clinical symptoms and signs, response to empirical and appropriate antibiotic and relevant investigations where available.

The first alert to the malabsorption was the failure to normalize TSH despite an incremental increase in thyroxine dosage to above 1.6 μg/kg/day [11], the general dose required dose to achieve euthyroidism. Additionally, the biochemical parameters were suggestive of the condition and this was further supported by the failure of serum thyroxine to rise following the 1 mg-thyroxine absorption test. The low serum protein including albumin and presumably transthyretin (pre-albumin) would avail less binding sites for the absorbed thyroxine and thus a relatively higher level of free (active) thyroxine to exert its effect upon peripheral tissues. Thus, this may have moderated the degree of hypothyroidism symptomatically. A trial of lio-thyronine (synthetic tri-iodothyronine) would have provided additional evidence of malabsorption. The lowering of TSH concentration at considerably higher doses of thyroxine suggested there was some absorption, probably from the large bowel [12]. However, as most of the thyroxine is absorbed from the ileum, this is consistent with the fact that WD commonly involves all portion of the small bowel and rarely the colon. This was the primary reason for the lack of response to thyroxine supplement.

It is highly probable that the WD putatively involved the thyroid as evident by the concomitant presence of hypothyroidism, the absence of thyroid auto-immunity and the recovery of normal thyroid function following antibiotic therapy, albeit with reduced thyroid reserve (*see below*). Further evidence stems from the fact that TW has been detected in many extra-intestinal organs ranging from cardiac valves, synovium, lung and pituitary [13-16] resulting in the corresponding clinical symptoms. In addition, the thyroid volume was slighted increased [1], suggesting the presence of oedema and inflammation. The thyroid nuclear uptake scan was inconclusive however. The seeding of the thyroid with TW is likely to be haemotogenous in nature given the TW PCR positivity in circulating mononuclear cells (MNC). Evidence of antibiotic effectiveness was also evident by the clearance of TW from circulating MNC and presumably thyroid tissue. It is appreciated however that the correlation between systemic Whipple's disease and its PCR positivity is far from perfect and the use of MNC PCR to monitor response to therapy remains controversial at this stage [15,17]. It is also critical that the diagnosis of TW-induced hypothyroidism was one of exclusion after other causes of hypothyroidism have been adequately excluded including negative/normal thyroid autoantibody studies including TSI. Ord's disease can cause transient hypothyroidism in the presence of negative anti-TPO and anti-Tg but the duration of hypothyroidism was probably too long for this to be the case. Also, the autoantibody titres mentioned remained unchanged overtime.

The gold standard for a reassessment of the condition would be a re-examination of the small bowel tissue for the clearance of the organism and resumption of normal bowel architecture. The patient, not unreasonably however, declined a repeat endoscopy due to a marked improvement in clinical response to antibiotic. It should be noted that WD and its characteristic PAS-positive granules on biopsy can remain positive for years after the treatment has been completed [15]. Presumably then duodenal tissue PCR for TW also remains positive for the same reasons, illustrating the discrepancy between clinical and histopathologic response. It remains to be seen if these two entities represent an unviable or simply dormant organism.

Although TW has been reported to involve the central nervous system in up to 90% of cases at postmortem examination [18], no such assessment was considered in this case due to the absence of neurological signs and excellent clinical progress. Agreeably, a proportion of WD involving the CNS will go undiagnosed [19].

Although the infection plausibly involved the thyroid, destruction must have been incomplete for euthyroidism to return. There might have been oedema and partial damage, impairing thyroid follicular function but with enough residual tissue to recover to euthyroidism. Previous postmortem report found complete obliteration of the thyroid gland with fibrous tissues [20]. There were foci of lymphocytes and cells containing PAS granules, which were not detected albeit in the FNA sample in this report. Contrary to intestinal villi from the gastrointestinal tract, which could be readily regenerated, thyroid tissues are not and thus it is assumed that some, but not all thyroid tissues are destroyed by the infective process. This is consistent with a previous case report [16] in which there was permanent panhypopituitarism despite the clearance of TW (as performed by PCR) after 4 weeks of appropriate antibiotic. Unfortunately and despite repeated attempts, direct evidence of TW involving the thyroid could not be obtained either by direct culture or PCR.

In order to assess thyroid reserve at the end of therapy, the TRH stimulation test was performed and revealed an exaggerated TSH response indicating impaired thyroid reserve [21,22]. This test was performed 18 months after the initial diagnosis of primary hypothyroidism to ensure that thyrotrophic cells have recovered from hyperplasia that can persist for weeks to months in primary hypothyroidism, even in the face of subsequent short-lived exogenous thyrotoxicosis. Thyrotrophic hyperplasia needed to be excluded because this can cause an augmented TRH-stimulated response in hypothyroidism and therefore may not reflect the patient's true thyroid status. In the absence of any other factors that may impair thyroid reserve, including smoking, iodine depletion and subclinical autoimmune thyroiditis, the demonstrated and diminished thyroid reserve can be attributed to TW infection. The patient is, of course, at risk of rapid and progressive hypothyroidism when the abovementioned risk factors intervened and hence requires frequent monitoring. As the TRH tests are unchanged and her TSH levels have remained normal so far, it can be deduced with confidence that the destructive inflammatory process incited by the presence of TW has been arrested but the damage permanent with a reduced thyroid reserve.

## Conclusion

The case highlights thyroid involvement by TW, causing significant thyroid failure. Despite the evidence of TW present in the duodenum and circulating leucocytes, its presence in thyroid tissue could not only be indirectly proven. However, the successful reversibility of the primary hypothyroidism in response to long-term anti-biotic therapy indicates strongly that WD indeed involves the thyroid gland in the absence of alternative causes. The damage to the thyroid has also resulted in permanent and reduced thyroid reserve as demonstrated by repeated TRH stimulation tests but with normal thyrotropin levels. This is only the second report that convincingly describes the putative link between primary hypothyroidism and WD and the first in a live patient with excellent recovery.

## Competing interests

The author(s) declare that they have no competing interests.

## Pre-publication history

The pre-publication history for this paper can be accessed here:


